# Further Investigations on Natural and Synthetic Bis-(1,2,3,4-tetrahydroisoquinoline) Derivatives Interacting with the Apamin-Sensitive Site of Small-Conductance Calcium-Activated Potassium Channels

**DOI:** 10.3390/molecules31152723

**Published:** 2026-08-05

**Authors:** Hossein Taouba, Romain Vitello, Donna Pereira Barbon, Jean-Luc Hayen, Coralie Herbet, Szymon Piotr Faliński, Nikolay Tumanov, Johan Wouters, Jean-François Liégeois

**Affiliations:** 1 Laboratory of Medicinal Chemistry, Center for Interdisciplinary Research on Medicines (CIRM), University of Liège, 4000 Liège, Belgiumcoralie.herbet@student.uliege.be (C.H.);; 2 Namur Institute of Structured Matter (NISM) and Namur Research Institute for Life Sciences (NARILIS) Research Institutes, Department of Chemistry, University of Namur, 5000 Namur, Belgium

**Keywords:** K_Ca_2.2, K_Ca_2.3, SK channels, affinity, binding, 1,2,3,4-tetrahydroisoquinoline, *in silico*, pharmacophore, alkaloids

## Abstract

Our previous study demonstrated that bis-(1,2,3,4-tetrahydroisoquinoline) (bis-THIQ) compounds such as tetrandrine and AG525E1 exhibit significant affinity for SK channels. Interestingly, the stereoisomers of AG525 display markedly different affinities toward SK channels, with the (*S,S*) stereoisomer being the best ligand. Previous structure-based *in silico* investigations failed to provide a satisfactory explanation for these differences. Moreover, a series of simplified THIQ derivatives derived from AG525 showed little or no affinity for SK channels, raising questions about the structural features required for binding to SK channels. To better understand the structure–activity relationships within this chemical family with diverse three-dimensional features, a ligand-based approach is developed in this work. Using a dataset composed of AG525 stereoisomers and natural bis-THIQ derivatives, a pharmacophore model was generated to identify key molecular features required for binding to SK channels and to rationalize the observed differences in activity. Twenty pharmacophore hypotheses were generated, with the best-ranked models highlighting five major interaction features: an aromatic ring, one acceptor group associated with this ring, one lipophilic feature, one positive charge, and a second acceptor site located farther from the THIQ moiety. This model differs from previously proposed pharmacophores emphasizing the presence of two positive charges. Two alkaloids recently identified as SK channel ligands, **(1)** and **(2)**, were found to match all five pharmacophoric features despite not being included in the training set. The absence of SK affinity of a series of simplified THIQ analogues derived from AG525 could be explained using the model. To go further, the predictive value of the model was evaluated by evaluating *in silico* the propensity of a series of cyclic and non-cyclic bis-THIQ alkaloids to bind to SK channels. High fitting scores were obtained for these compounds, and subsequent *in vitro* experiments, using radioligand competitive binding assays, were performed and confirmed that they belong as ligands.

## 1. Introduction

**Figure 1:**
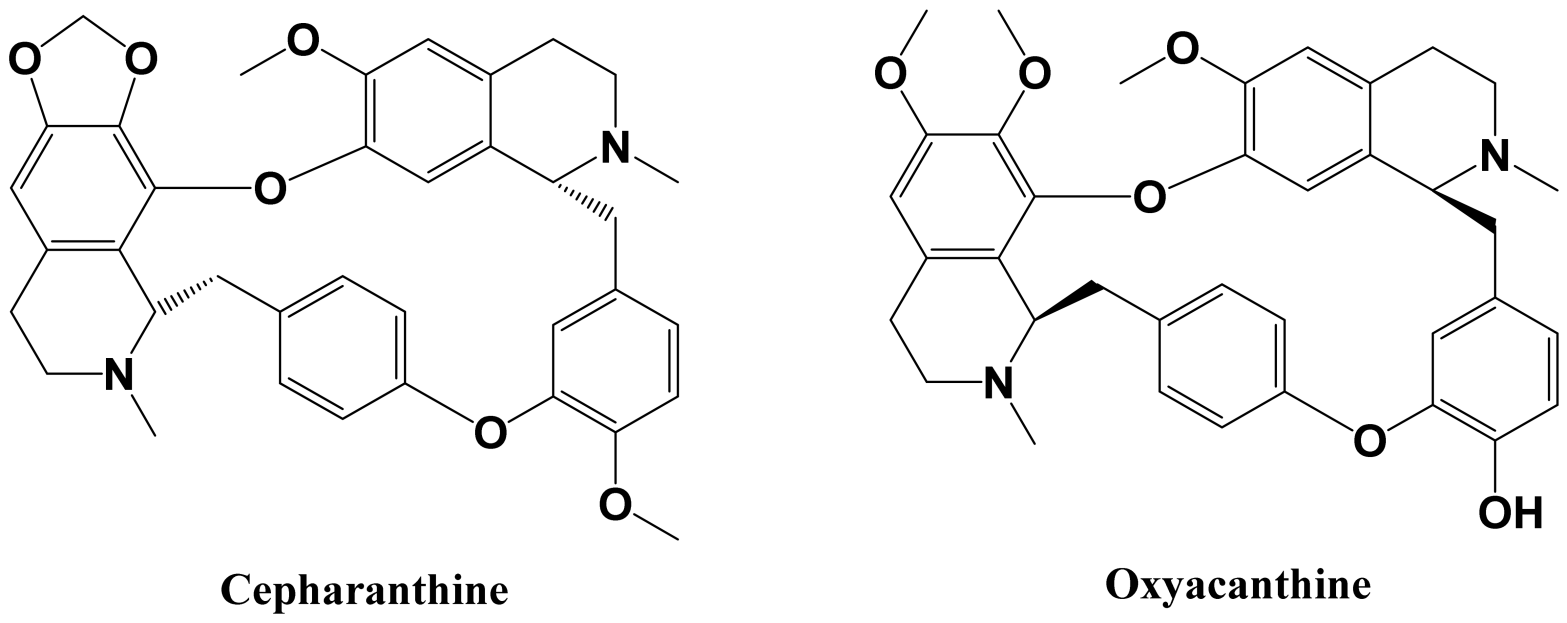
Chemical structures of two recently identified SK channel ligands [3,4].

Bis-(1,2,3,4-tetrahydroisoquinoline) derivatives (Bis-THIQ) represent a promising class of natural alkaloids in terms of their biological and therapeutic potential [1,2]. The affinities of two compounds from this class, cepharanthine **(1)** and oxyacanthine **(2)** (Figure 1), for small-conductance calcium-activated potassium (SK) channel subtypes were recently reported by our group [3].

SK channels are voltage-independent membrane proteins that open in response to increases in intracellular Ca^2+^ concentration. Although they are K^+^ ion permeable, their conductance is lower than that of other potassium channel types. These characteristics make them ideal candidates for regulating several physiological mechanisms. Primarily, they modulate neuronal firing rate by contributing to the AfterHyperPolarization (AHP) phase of the neuronal refractory period [5]. Peripherally, they are also expressed in the cardiac atria, where they are involved in heart rate regulation [6].

Thus, various tissues can differentially express one or more of the three known SK channel subtypes: SK1 (K_Ca_2.1), SK2 (K_Ca_2.2) or SK3 (K_Ca_2.3) (encoded by *KCNN1*, *KCNN2* and *KCNN3* genes, respectively). SK channel subtypes are implicated in numerous pathologies, often with a specific mechanism linked to a single subtype. Prominent examples include depression and other mood disorders, where SK3 channels are upregulated in serotonin-producing neurons of the basal nuclei [7,8], and atrial fibrillation, which is associated with SK2 overexpression in atrial cardiomyocytes [6]. Both subtypes are also upregulated during the progression of various cancers [9,10]. Specifically, the SK2 subtype is expressed under hypoxic conditions, such as in melanomas [11], whereas the SK3 subtype drives the mobility of several cancer cell types, including invasive breast cancer [12].

**Figure 2:**
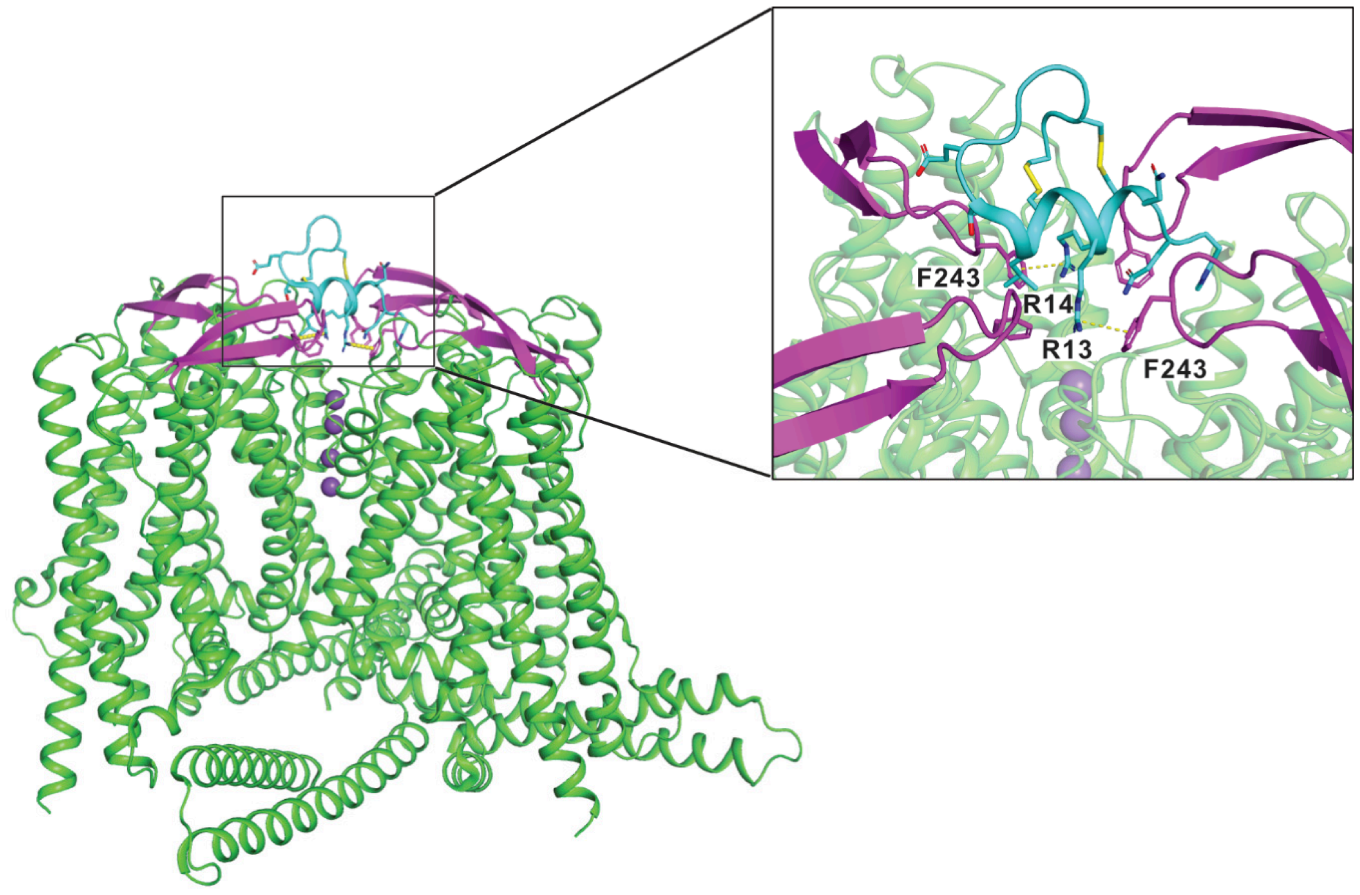
Structural basis of apamin recognition by the human SK2 channel (PDB ID: 9VUA) [13]. Cartoon side view of the human SK2 channel in complex with apamin. SK2 is shown in green, apamin in cyan, and the extracellular S3–S4 linkers contributing to the apamin-binding vestibule in magenta. The boxed region is enlarged in the right panel to highlight the binding interface. The structured S3–S4 linkers project toward the central pore axis and position F243 within the extracellular vestibule. The side chains of apamin residues R13 and R14 insert between F243 residues from adjacent SK2 subunits, forming cation–π interactions indicated by yellow dashed lines. These interactions contribute to the high-affinity recognition and pore-blocking action of apamin.

Cartoon side view of the human SK2 channel in complex with apamin. SK2 is shown in green, apamin in cyan, and the extracellular S3–S4 linkers contributing to the apamin-binding vestibule in magenta. The boxed region is enlarged in the right panel to highlight the binding interface. The structured S3–S4 linkers project toward the central pore axis and position F243 within the extracellular vestibule. The side chains of apamin residues R13 and R14 insert between F243 residues from adjacent SK2 subunits, forming cation–π interactions indicated by yellow dashed lines. These interactions contribute to the high-affinity recognition and pore-blocking action of apamin (Figure 2).

Consequently, these properties can be exploited to develop novel therapeutic approaches targeting SK channels for the treatment of such pathologies. A prominent example involves the cardiovascular system, where an SK2 channel modulator is currently under advanced preclinical investigations for its potential to protect against atrial fibrillation by selectively targeting cardiac SK channels [14,15,16].

The most potent SK channel ligands are toxins, with apamin having long served as the reference compound [17,18,19]. Structurally, apamin is an 18-amino-acid peptide stabilized by two disulfide bridges into a rigid, globular conformation, featuring a short α -helix and a β -turn [19,20,21]. This small peptide from bee venom displays very high affinity for all SK channel subtypes, without marked subtype selectivity, thereby conferring specificity for the SK family. Apamin binds to the outer pore region [13,22,23,24,25], and its radioiodinated analog is used for *in vitro* competitive binding assays [26,27].

**Figure 3:**
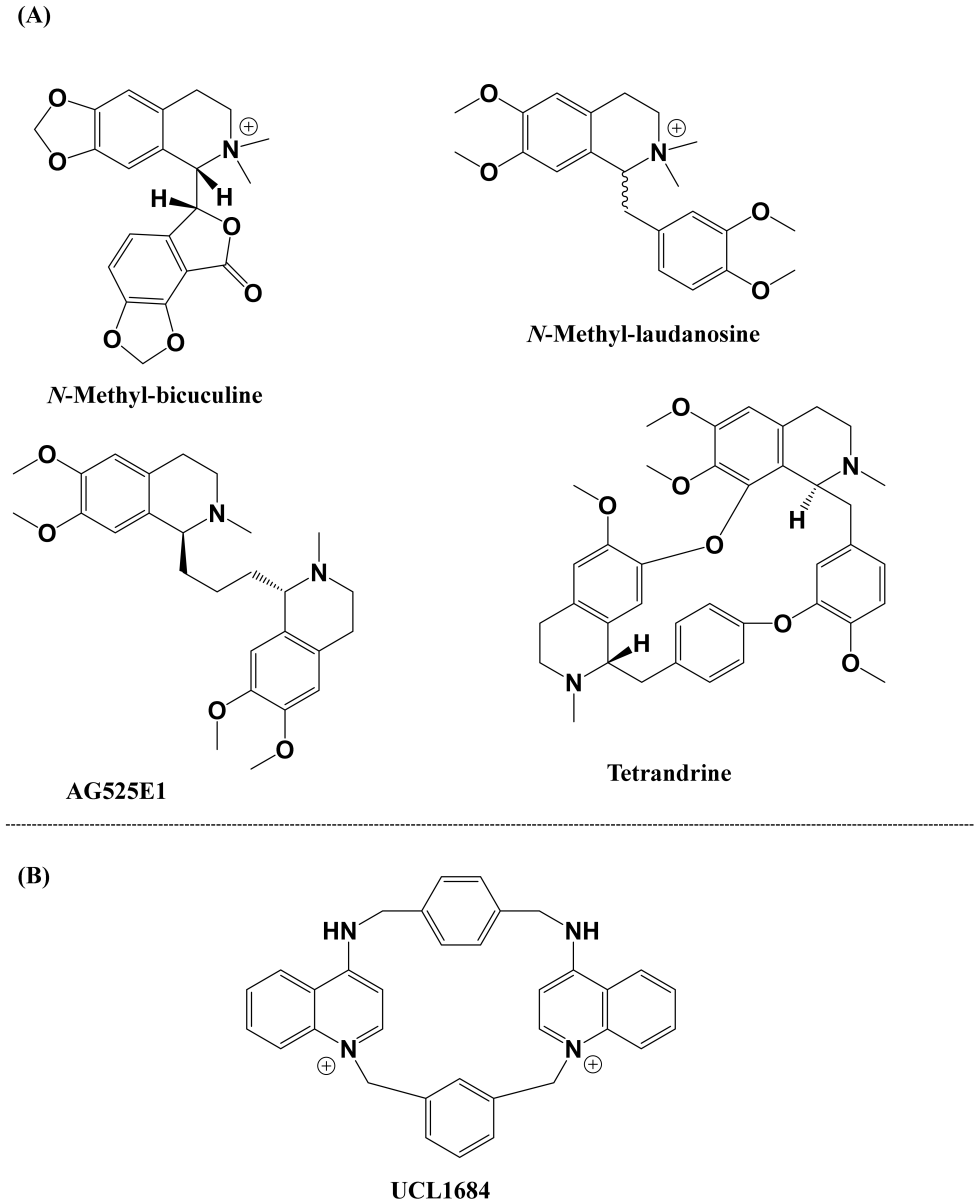
Chemical structures of five of the previously identified SK channel ligands targeting the apamin-binding site. (**A**)-Isoquinoline derivatives (**B**)-Quinoline derivative.

However, despite its high potency, the peptidic nature of apamin inherently limits its clinical utility due to poor oral bioavailability and low blood–brain barrier permeability [28,29,30]. This shifted research interest toward low-molecular-weight, non-peptidic alternatives capable of drug-like development. Various non-peptidic SK channel blockers have been developed to target the apamin-sensitive site, with two main chemical series represented by quinoline and isoquinoline derivatives. Within the former group, the bis-quinolinium structure UCL1684 (Figure 3) has been extensively studied in the literature [22,23,31]. Within the latter, *N*-methyl-bicuculline, a 1,2,3,4-tetrahydroisoquinolinium derivative (Figure 3), represents the earliest SK ligand that was discovered in our program [32]. Optimization of this scaffold first led to *N*-methyl-laudanosine [33], and subsequently to a bis-THIQ series, from which AG525E1 emerged as the most potent basic small-molecule SK blocker to date (Table S2, see Supporting Information) [34]. AG525E1 is the (*S*,*S*)-enantiomer of a diastereoisomeric mixture. It exhibits superior affinity compared to its stereoisomers, highlighting the importance of chirality, although the structural basis for these differences could not be resolved by *in silico* investigations [35]. Owing to its basic nature, it crosses the blood–brain barrier and alters dopamine release *in vivo* [36,37]. This molecule was selected as the basis for investigating the structural characteristics required for a THIQ molecule to interact with the apamin-sensitive site of SK channels.

A previous pharmacophore model [38], based on apamin and UCL1684, revealed the necessity for two positive charges (arginine-13 and -14 in apamin and the two quinolinium nitrogens in UCL1684) separated by a distance of ~5.6 Å. While these pharmacophoric elements accounted for the affinity of a series of bis-quinolinium derivatives [39], they failed to explain that of the bis-THIQ analogs, in particular the importance of chirality [35]. In earlier studies [27,39,40,41], THIQ moiety substitutions were investigated. Firstly, the quaternized and positively charged analogs, namely the tetrahydroisoquinolinium (THIQ+) analogs, are more potent than the basic and transiently charged THIQ compounds [27,40]. For the quaternization of the nitrogen atom at this position, a methyl substituent yields better results than longer chains such as ethyl, butyl and benzyl groups [27,40]. Moreover, small alkyl substituents at position 1 of the heterocycle reduce the SK affinity of laudanosine derivatives, while 3,4-dimethoxybenzyl and 2-naphthylmethyl substituents both increase it [27,39,41]. Finally, adding methoxy groups at positions 6, 7, or 8 of the heterocycle increases the potency of the compounds as SK ligands, whereas small alkyl groups as well as halogens at these same positions decrease it [27,39,40,41].

Based on this, we aimed to design simplified derivatives—particularly regarding chirality—to reduce the number of stereoisomers and identify the minimal scaffold. Consequently, a series of mono-THIQ analogs possessing a flexible basic side chain was synthesized [4]. This new series of SK channel ligands simplifies the AG525 structure by pairing a single THIQ moiety with an amine chain to mimic apamin, maximizing synthetic yields to 50% per enantiomer through reduced chirality. To optimize potency, the design explores various methoxylation patterns on the aromatic ring to enhance binding affinity (Figure S2; see Supporting Information). Their enantiomers were resolved and evaluated for their affinity toward SK channels. Unfortunately, these compounds exhibited no affinity for the apamin-sensitive site of SK channels [4].

Based on these developments and the collected data, we aimed to reexamine these structures, hoping to generate a new pharmacophore model. The binding prerequisites for SK channels were first recalculated using a ligand-based approach, where the calculations were guided by the structures and affinities of known ligands. The models were generated by the Schrödinger Maestro Phase software suite (see Supporting Information). The training set consisted exclusively of bis-THIQ derivatives to ensure the resulting models were specifically tailored for this class of compounds. More precisely, the Ki values of the series of AG525 isomers, as well as tetrandrine, a natural bisbenzylisoquinoline alkaloid (Figure 3) that exhibits an affinity of 0.306 ± 0.050 µM for SK2 channels and 0.461 ± 0.042 µM for SK3 channels [35], and their quaternized *N*,*N′*-dimethyl analogs were utilized [35]. Finally, the crystal structure of AG525E1 [42] was selected to generate stable starting conformations for all related analogs (see Table S2 and Figure S4, Supporting Information).

**Figure 4:**
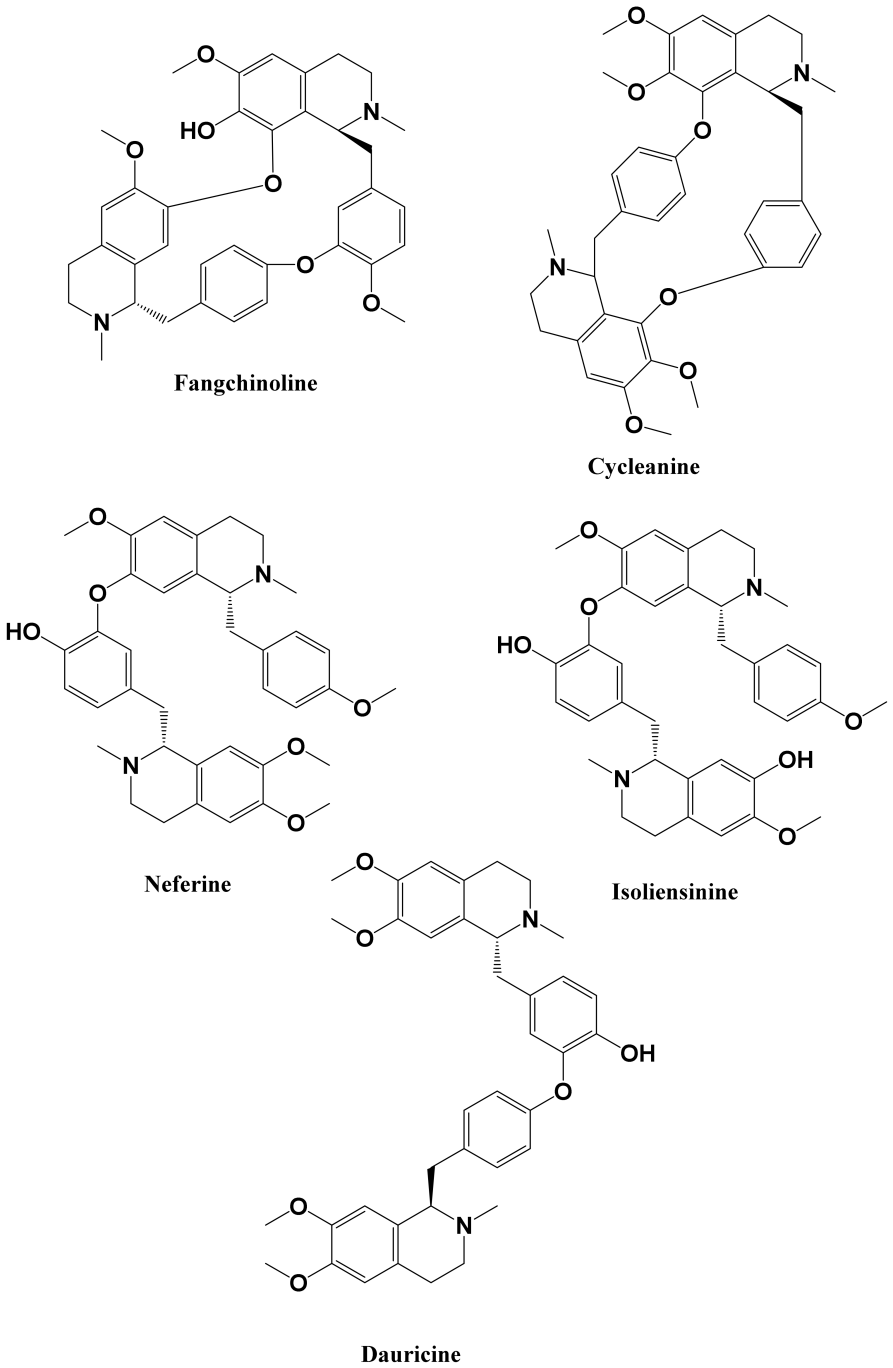
Chemical structures of cyclic and non-cyclic bis-THIQ alkaloids evaluated as potential SK channel ligands using the *in silico* approach.

To thoroughly validate the predictive power of this new pharmacophore model, we decided to screen our natural product library. This library includes **(1)** and **(2)** [3], alongside newly acquired derivatives such as dauricine **(3)**, neferine **(4)**, isoliensinine **(5)**, fangchinoline **(6)**, and cycleanine **(7)** (Figure 4).

**(3)** has been studied for its neuroprotective [43] and anti-inflammatory effects [44,45,46,47], while **(4)** has shown potential as both an anti-tumor agent [48,49,50] and a cardioprotective compound [51]. **(5)** has primarily been investigated for its synergistic anti-cancer activity and antioxidant properties [50,52]. Meanwhile, **(6)** has been studied for its anti-metastatic [53,54] and antiviral activities [55], whereas **(7)**, a calcium antagonist [56], exhibits antiplasmodial and apoptotic activities [57,58]. These alkaloids, alongside being evaluated *in silico*, were subsequently assessed in our radioactive binding procedure using two different types of preparations: one containing SK2 channels and the other SK3 channels, with ^125^I-apamin as the radioligand. This enabled us to determine their binding affinities for SK2 or SK3 channels and their subtype selectivity.

This structural diversity within the screened library provides an opportunity to investigate how the nature of the linkages connecting the two THIQ moieties influences affinity for SK channels. In particular, comparing cyclic and acyclic bisbenzylisoquinolines, as well as the different diphenyl ether connectivity patterns, may help identify the structural arrangements that most favor interaction with the apamin-sensitive site of SK channels.

## 2. Results

Twenty models were generated to identify pharmacophoric features that describe the potential interaction points shared by the selected ligands. These models, consisting of four or five points, were ranked based on their scoring functions (Table S1; see Supporting Information).

Several observations can be made:

(i) The top three models (Figure S1) are highly similar in terms of pharmacophoric features. They describe nearly identical interactions located within the same region of the molecule. It shows one of the top five-point models, combining pharmacophoric features (R: aromatic, P: positive, H: hydrophobic, and A: H-bond acceptor), superimposed onto one of the molecules from the training set.
(ii) Interestingly, according to the models, a second positive charge does not appear to be mandatory for SK affinity. None of the twenty models included a second positive charge as one of the pharmacophoric interaction points, although each reference ligand used in this study possessed two charged or ionizable nitrogen atoms (Figure S1; see Supporting Information). This finding contrasts with previous pharmacophore models, which highlight the presence of two charged nitrogen atoms separated by a distance of 5.6 to 5.8 Å [38,59]. This discrepancy may explain the lack of affinity observed for a series of simplified THIQ derivatives recently synthesized [4].

**Figure 5:**
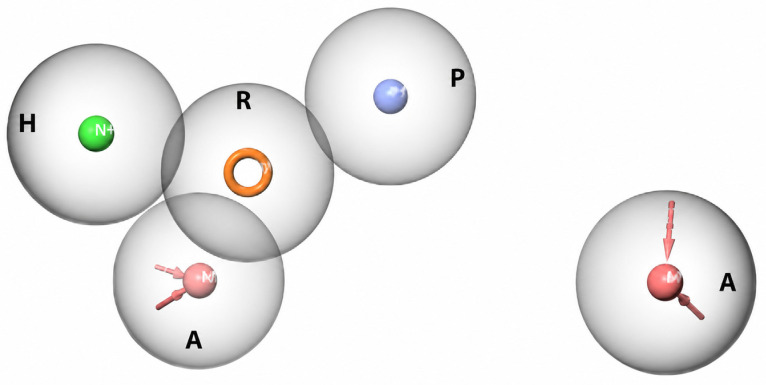
Pharmacophore model selected for the study (AAHPR_2) illustrating its key features. Feature types are defined as follows: A = H-bond acceptor (red dot), H = hydrophobic (green dot), R = aromatic (orange circle), P = positive (blue dot). (more details in Table S1 and Figure S1).

**Figure 6:**
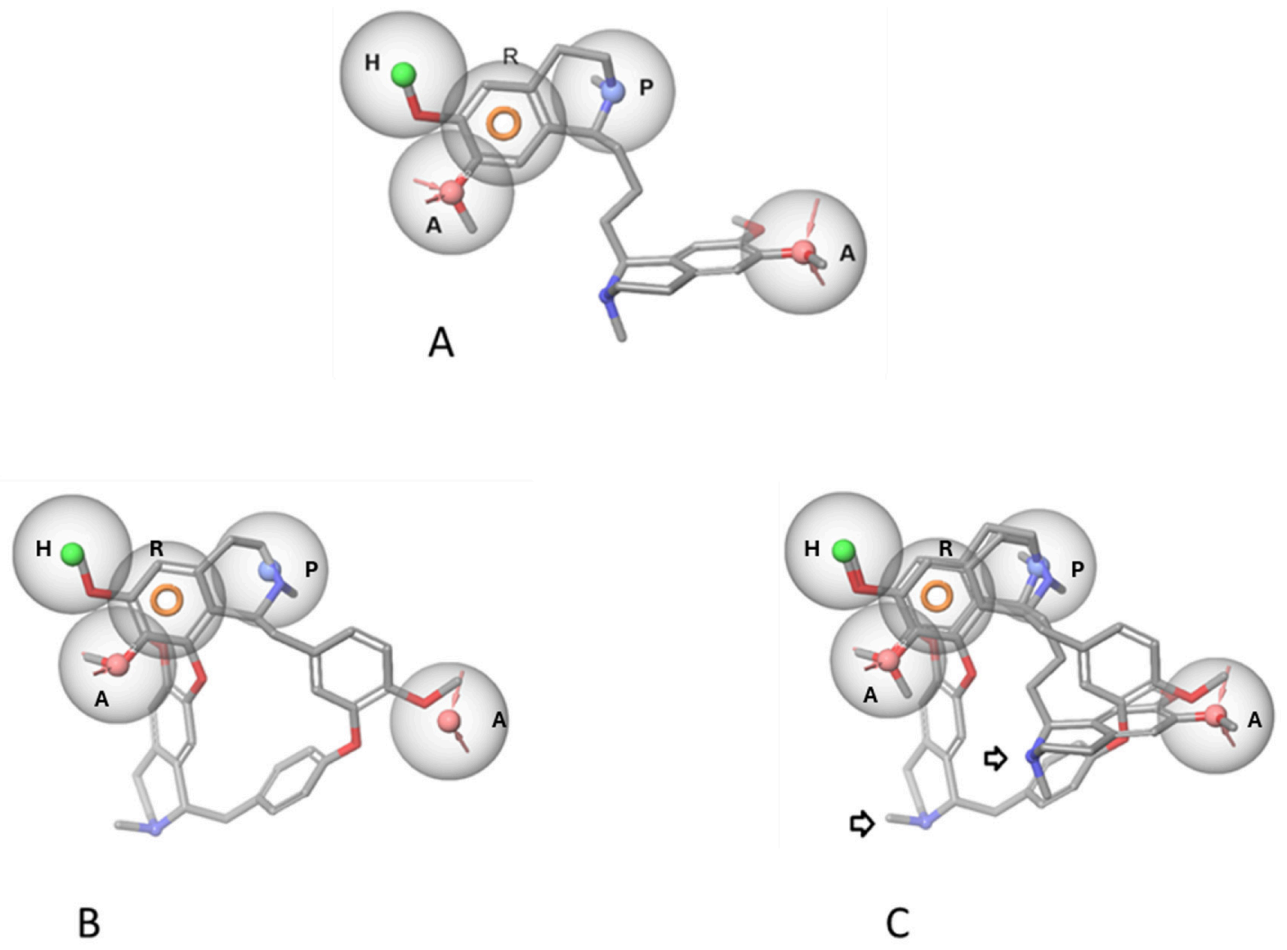
Representation of the selected pharmacophore model associated with bis-THIQ molecules interacting with the apamin-sensitive site of SK channels. (**A**) Superimposition of AG525E1 with the pharmacophore model; the five pharmacophoric features (R: aromatic, P: positive, H: hydrophobic, and A: H-bond acceptor) are aligned with the corresponding chemical groups of the compound. Note that the methoxy substituents on the aromatic ring can be associated with either a hydrophobic (H) or H-bond acceptor (A) anchoring point, explaining the similarity between the top three generated models (i.e., AHHPR and AAHPR). (**B**) Superimposition of tetrandrine with the pharmacophore model. (**C**) Superimposition of both compounds, AG525E1 and tetrandrine, with the pharmacophore model. The arrows indicate the N atom in both compounds that do not overlap and therefore do not constitute a common pharmacophore site.

To further describe the most prominent models (Figure 5), the potential ligands appear to require an aromatic ring substituted with one positively charged group (P), and two features that can act either as hydrophobic (H) or hydrogen bond acceptor (A) depending on the model. These features essentially correspond to two methoxy groups substituting the ring, with the models focusing either on the methyl moiety or the oxygen atom of each substituent (Figure 6A). Therefore, since each methoxy substituent on the aromatic ring can be associated with either a hydrophobic (H) or an electron acceptor (A) anchoring point, the strong similarity observed between the top three generated models (e.g., AHHPR and AAHPR) can be readily explained. In addition to these pharmacophoric elements, the structure also appears to require a hydrogen bond acceptor site located farther away from the other interaction points. Finally, none of the generated five-point pharmacophores contained two positively charged elements (P).

The correspondence between the chemical structure of AG525E1 and the proposed interaction points of the selected pharmacophore model (AAHPR) is shown in Figure 6A, while the corresponding representation for tetrandrine is shown in Figure 6B. The second quaternized or protonated nitrogen atom of the different ligands used to generate the pharmacophore was found at clearly distinct locations (Figure 6C), suggesting that it is not an essential pharmacophore feature for this series of molecules.

In this model, four points (AHRP) correspond to four sites located on a single THIQ ring in both AG525E1 and tetrandrine, while the fifth point (A) corresponds to a methoxy group of the second THIQ ring of each compound. The superimposition of both molecules shows that the second basic nitrogen atom (arrows in Figure 6C) does not overlap.

**Figure 7:**
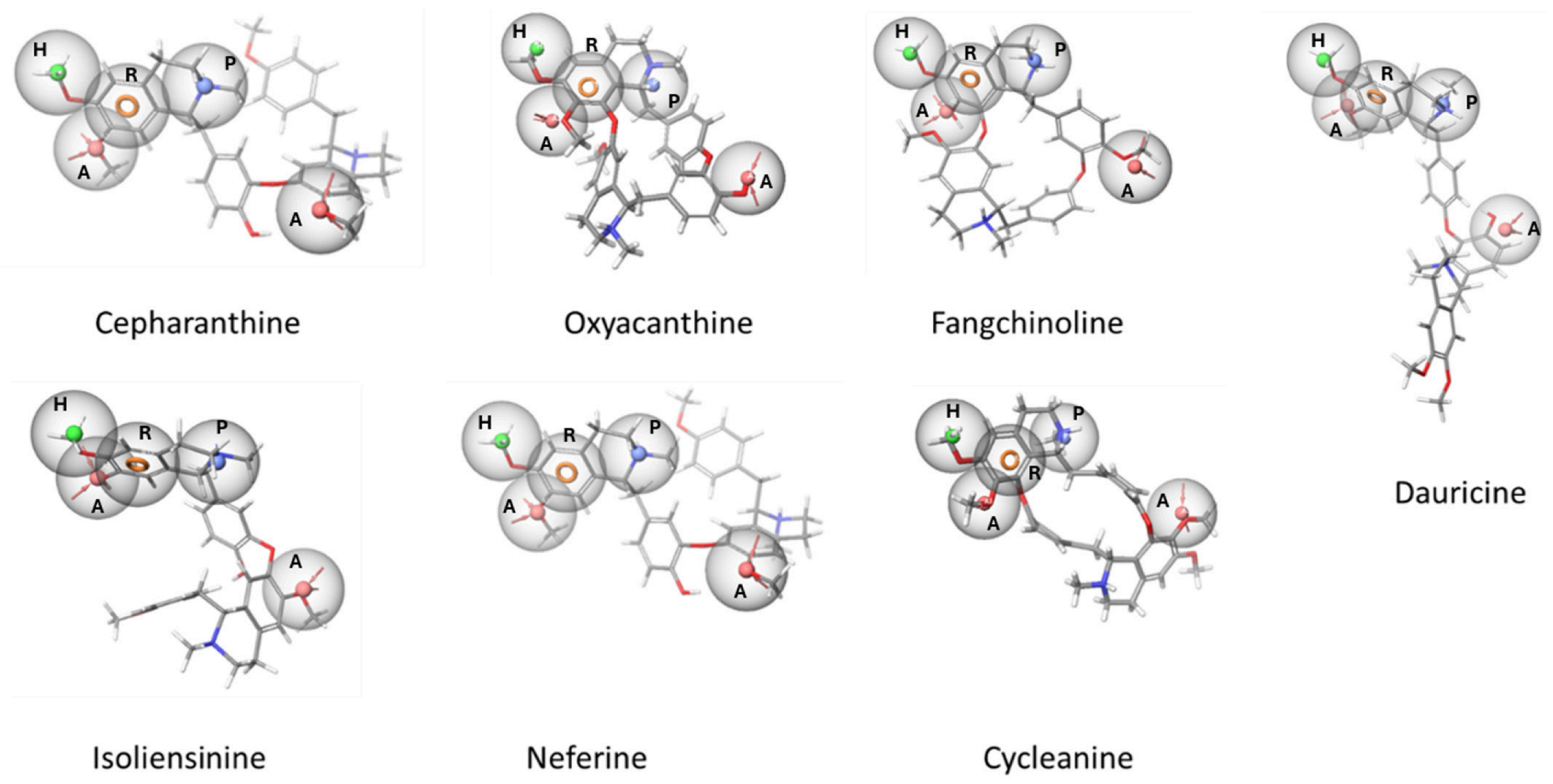
Superimposition of newly reported bis-THIQ alkaloids exhibiting affinity for the apamin-sensitive site of SK channels with the selected pharmacophore model.

The significance and predictive value of the best-fitting model obtained in this study were then further investigated. The pharmacophore hypothesis was superimposed onto the structures of the recently published SK ligands **(1)** and **(2)** [3]. These cyclic bis-THIQ alkaloids (Figure 1), commonly used therapeutically [1,2], were recently shown to exhibit micromolar to submicromolar affinity for the SK2 and SK3 subtypes (Table 1). The superimposition of the 3D structures of these alkaloids with the AAHPR model revealed that, despite being absent from the training dataset, both **(1)** and **(2)** matched all five proposed pharmacophoric points through corresponding chemical groups, highlighting a similar spatial topology of these features (Figure 6). Similarly to AG525E1 and tetrandrine (Figure 6), **(1)** and **(2)** (Figure 7) concentrate four interaction points on a single THIQ ring, while the fifth point corresponds to either a methoxy group on the second THIQ ring in **(1)** or to the phenolic group of a linker connecting both THIQ rings in **(2)**.

Otherwise, the model was also used to investigate the lack of affinity observed for a series of simplified THIQ derivatives recently developed [4]. These compounds contain a single THIQ ring and a flexible side chain of variable lengths bearing a distal basic group. Different stereoisomers were evaluated using this *in silico* model. The results showed that these simplified structures failed to adequately match the five-point model (Supplementary Figure S3) [4].

The new model is able to predict the affinity of **(1)** and **(2)** for SK channels, although these compounds were not included in the dataset used to generate the model. This prompted us to further evaluate the predictive power of the pharmacophore by investigating additional molecules. Accordingly, several bis-THIQ alkaloids, namely **(6)**, **(4)**, **(5)**, **(3)** and **(7)** (Figure 5), were selected. **(6)** is structurally the closest to the previously evaluated alkaloids, displaying a cyclic bis-THIQ scaffold, while **(7)** is also a cyclic bis-THIQ derivative but contains a markedly different linkage between the two THIQ rings compared with other cyclized derivatives (Figure 5). This structural particularity confers greater rigidity to these molecules. In contrast, **(3)**, **(5)** and **(4)** exhibit non-cyclic and less rigid structures (Figure 5) with different connectivities between the two THIQ rings. Consequently, these compounds are more flexible and can adopt a broader range of spatial conformations due to their higher degree of freedom.

These bisbenzylisoquinoline alkaloids consist of two benzylisoquinoline (BI) subunits linked by ether bridges. In addition to these ether linkages, methylenedioxy bridges or direct carbon-carbon bonding may connect the monomeric units. Structurally, these molecules are classified based on variations in: (1) the number of aromatic oxygen substituents; (2) the number of ether linkages; (3) the type of ether bridges (i.e., diphenyl ether or benzylphenyl ether); and (4) the attachment sites on the BI units for the ether or carbon-carbon bonds. [60,61]. It is also interesting to look at the specific absolute configurations at the C-1 and C-1′ chiral centers [62]. The acyclic derivatives possess a single diphenyl ether linkage with a (1*R*,1′*R*) [62,63] configuration, exemplified by **(3)** (a type I bisbenzylisoquinoline with an 11–12′ linkage), as well as **(4)** and **(5)** (type V bisbenzylisoquinolines with an 11–7′ linkage). Conversely, the cyclic alkaloids incorporate two diphenyl ether linkages; these include **(1)** and **(2)** (type VI, featuring 7–8′ and 11–12′ linkages, with a (1*R*,1′*S*) configuration [62]), tetrandrine and **(6)** (type VIII, featuring 8–7′ and 11–12′ linkages, with a (1*S*,1′*S*) configuration [62,64]), and **(7)** (type XX, featuring 8–8′ and 12–12′ linkages, with a (1*R*,1′*R*) configuration) [60,61,62].

When aligned with the pharmacophore model (Figure 7), these compounds were able to match each interaction site with the corresponding chemical group, suggesting a potential interaction with SK channels. Although **(3)**, **(5)** and **(4)** possess less rigid structures than **(1)**, **(2)** and **(6)**, they display four interaction points clustered around one THIQ ring, while the fifth point is provided by a methoxy group located on the second THIQ ring (Figure 6). These non-cyclic compounds also exhibit distinct orientations of the remaining parts of their structures. Although the more flexible regions of the molecules adopt conformations that differ substantially from those observed in cyclic alkaloids, the key interaction points maintain a comparable and close spatial arrangement. This observation suggests that, despite adopting spatial conformations distinct from those of cyclic bis-THIQ alkaloids, these new candidates still possess a favorable profile for interaction with the SK channel.

**Figure 8:**
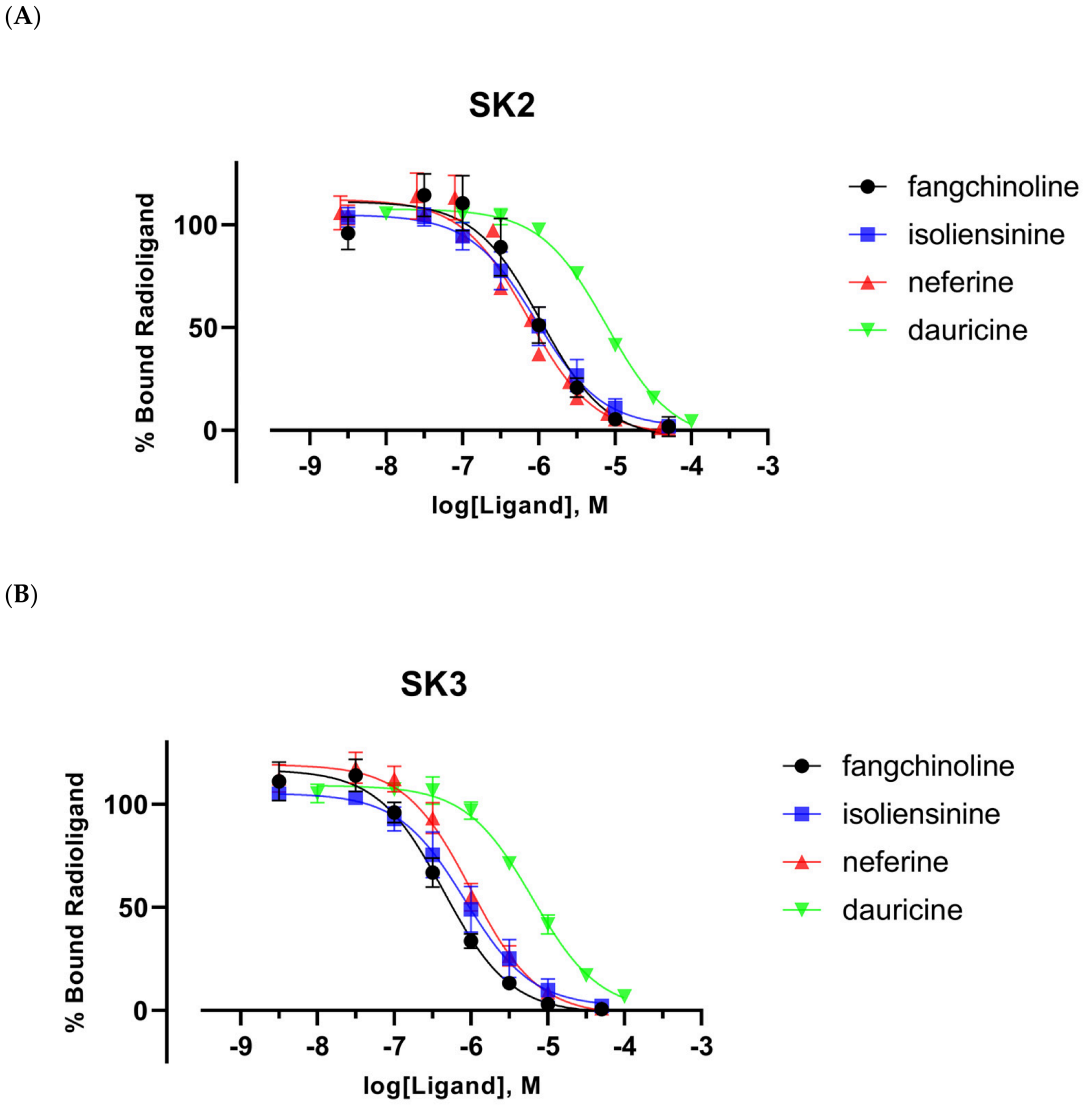
Concentration-response curves for the displacement of ^125^I-apamin by bis-THIQ alkaloids on (**A**) SK2 and (**B**) SK3 channel subtypes. Data points represent mean ± SD from independent experiments performed in duplicate for each concentration (the exact number of experiments for each compound is detailed in Table 1).

The *in silico* investigation was followed by experimental evaluation of these molecules using the same *in vitro* binding assay employed for the other SK ligands. The affinities of these compounds are reported in Table 1. **(6)**, **(5)** and **(4)** displayed submicromolar affinity for both the SK2 and SK3 subtypes, ranking them among the most potent basic non-peptidic ligands of the apamin-sensitive site of SK channels (Figure 8). To date, all known ligands of the apamin-sensitive site of SK channels function exclusively as blockers, with no alternative mechanism of action documented in the literature [18,31,34,35,65,66]. **(3)** showed a slightly lower affinity in the micromolar range, whereas **(7)** did not significantly displace the ligand at 10^−5^ M during the screening experiments.

These results indicate differences in the ability of these alkaloids to interact with the apamin-sensitive site of SK channels. Bis-benzylisoquinolines bearing a single diphenyl ether linkage, whether in a tail-to-tail orientation (type I) or a head-to-tail orientation (type V), exhibit affinity for SK channels. The head-to-tail orientation (**(5)** and **(4)**) appears to be the most favorable for interaction with the apamin-sensitive site of the SK channel. It would therefore be particularly interesting to further explore the type V family of bisbenzylisoquinoline alkaloids, including subtypes Va–Vd, in order to determine whether differences in oxygenation patterns influence affinity for SK channels [67]. Furthermore, compounds containing two diphenyl ether linkages, one head-to-head and the other tail-to-tail (types VI and VIII), also display favorable affinity for SK channels. Based on our results, neither of these two families appears to be clearly favored. Interestingly, **(1)** and **(6)** both possess free phenolic groups, whereas tetrandrine and **(2)** are more extensively methoxylated and hydrophobic. Free phenolic groups seem to be less favorable for interaction with the SK channel, whereas methoxylation of this position seems to enhance affinity. Such differences in oxygenation patterns and conformational flexibility may also contribute to SK channel affinity and deserve further investigation (Figure 7).

**(7)** was the only bisbenzylisoquinoline in this study that failed to bind to the channels, despite being predicted by the model to possess SK channel affinity. This lack of affinity may be explained by the head-to-tail orientation of the linkage between its two THIQ moieties. Surprisingly, **(7)** shares a highly similar head-to-tail macrocyclic linkage framework based on two diaryl ether bridges with *d*-tubocurarine (7–12′ and 11–12′) [61,68]. It is recognized for its blocking activity on the apamin-sensitive site of SK channels [32,69,70]. The structural distinction lies in their nitrogen atoms, as *d*-tubocurarine possesses one permanently charged quaternized ammonium group rather than two tertiary amines. It would also be important to confirm whether two diphenyl ether linkages arranged in a head-to-tail orientation reduce affinity for SK channels by evaluating additional type XX bisbenzylisoquinoline alkaloids, as well as type XXI derivatives characterized by 8–12′ and 11–7′ head-to-tail diaryl ether linkages. Furthermore, stereoisomerism may also account for this result; in cyclic analogs, only the (1*R*,1′*R*) configuration lacks affinity for SK channels, whereas the (1*R*,1′*S*) and (1*S*,1′*S*) configurations are active. In contrast, for the acyclic series, only the (1*R*,1′*R*) isomers could be evaluated, leaving the biological activity of the remaining stereoisomers undetermined.

To further investigate the structural determinants of SK channel affinity, it would be particularly interesting to evaluate alkaloids containing a single diphenyl ether linkage in a head-to-head orientation (type IV), as well as compounds possessing two diphenyl ether linkages exclusively in a head-to-head orientation (types XVIII and XIX). Such studies could help identify the most favorable structural arrangement for interaction with SK channels.

The present ligand-based approach appears promising, as the generated pharmacophore model(s) were able not only to explain the experimental affinities observed for bis-THIQ derivatives, but also to predict the affinity of newly investigated alkaloids that, *a posteriori*, proved to be good ligands of the apamin-sensitive site of SK channels. The pharmacophore model also accounted for the lack of affinity observed with a recently developed series of simplified mono-THIQ derivatives [4].

This work therefore represents a first step toward a more rational design of ligands targeting this interaction site. In the future, additional studies could incorporate structure-based approaches using cryo-EM models of SK2 channels [13,24,25]. Combining ligand-based and structure-based approaches should facilitate the discovery and optimization of new SK channel ligands.

## 3. Material and Methods

### 3.1. Drugs

Compounds tested in this study were purchased from the following companies: fangchinoline (ABCR AB550543, Karlsruhe, Germany, 97%), isoliensinine (ABCR AB493998, 95%); dauricine BioConnect (TargetMol T6S0119, Boston, MA, USA, >99%); cycleanine (TargetMol T8206, >99.5%), neferine (Sigma-Aldrich SML0349, St. Louis, MO, USA, ≥98%). They have been used without further purification. The simplified AG525 analogs purity range between 91% to 99%. The compounds were dissolved in DMSO at 40 × 10^−4^ M with sonication before the experiments.

### 3.2. Experimental Procedures for Cell Culture and Transfection, Biological Material Preparation and Affinity Binding Measurements

#### 3.2.1. Cell Culture

Media, complements and consumables were purchased from different companies (VWR, Leicestershire, UK; Fisher, Pittsburgh, PA, USA; Thermo Scientific, Waltham, MA, USA; Sigma-Aldrich, St. Louis, MO, USA; Greiner, Kremsmünster, Austria; Biowest, Nuaillé, France; Gibco, Grand Island, NY, USA; Falcon, Corning, NY, USA, Eppendorf, Hamburg, Germany).

HEK293 cells were used. This cell line was available in the laboratory and was maintained using Dubelco’s Modified Eagle Medium (DMEM) containing high glucose, stable-glutamine and complemented with Foetal Bovine Serum (FBS) and a Penicillin-Streptomycin mix (100 U/L and 100 mg/L respectively) and was incubated at 37 °C and under 5% CO_2_ atmosphere. The cells were passaged twice a week with a 10% dilution. To do so, cells were detached by 5 min of trypsinization at 37 °C and were then centrifuged at about 250× *g* for 5 min at room temperature before resuspension in fresh medium. The HEK293 cells were kept for a maximum of 25 cycles.

#### 3.2.2. Transient Transfection

The cells were transiently transfected as needed using linear PolyEthyleneImine (M.W. 25,000 Da) from Alfa Aesar (Lancashire, UK) (ref. 43896.01) with plasmids containing the KCNN2 and KCNN3 human genes bought from Genscript (Nanjing, China). The genes were inserted in a pcDNA3.1 plasmid in house. The complete plasmids were therefore available at the laboratory.

#### 3.2.3. Biological Material Preparations

HEK293 cells were plated on 100 mm Petri dishes for 24 to 48 h, until a confluence of about 90% (75% to 100%) was reached. They were then transfected with plasmids containing either the hSK2 or the hSK3 gene, in a 0.2 µg/µL solution of PEI. Cells from 5 to 10 Petri dishes were harvested together after 48 h of contact by 5 min of trypsinization at 37 °C and concentrated by 5 min of centrifugation at about 250× *g* at room temperature. The pellet was then resuspended in 3 mL per dish of cold PBS (4 °C) and concentrated by 10 min of centrifugation at 1000× *g* at 4 °C. This PBS wash was repeated a second time. Afterwards, the pellet was resuspended in a lysis buffer (50 mM Tris, 1% BSA, pH 7.4), mixed thoroughly using a 21 G needle mounted on a syringe, and centrifuged at 200× *g* for 10 min at 4 °C. The supernatant was gathered and centrifuged for 25 min at 20,000× *g* at 4 °C, the resulting pellet was then resuspended in a storage buffer (50 mM Tris, 5 mM KCl, pH 8.5) using 1 mL per dish. The resuspension in storage buffer and centrifugation was repeated a second time.

The protein concentration was determined using a colorimetric protein assay kit (Pierce BCA protein assay kit, Rockford, IL, USA at Thermo Scientific). The absorbance of the samples and of a reference scale was measured on transparent 96-well plates at 560 nm on a FlexStation3 apparatus (Molecular Devices, San Jose, CA, USA). The concentration was calculated by linear regression using the scale. The preparation was then diluted with 10% (*v*/*v*) glycerol and aliquots were stored at −80 °C before use.

#### 3.2.4. Binding Experiments

The incubation buffer and the wash buffer had the same composition in this procedure and consisted of 10 mM of Tris-HCl (pH 7.5), with 0.1% (*m*/*v*) of BSA and 5 mM of KCl. The radioligand was ^125^I-apamin, with a specific activity of 2000 to 2200 Ci/mmol, and was used at a concentration of about 5 pM in the assay. Unlabeled apamin was diluted in mQ water at a concentration of 400 µM as stock solution and aliquots were stored at −20 °C before use. Glass fiber filters (Whatman GF/C, Cytiva, Marlborough, MA, USA) were pre-soaked in a 0.5% (*m*/*v*) PEI (Sigma-Aldrich P3143) solution and washed with 2.5 mL of ice-cold buffer right before filtration. A membrane preparation containing either cloned hSK2 or hSK3 channels at corresponding concentration of 10 µg of protein per mL was prepared in the buffer. Membrane samples of 950 µL were then incubated for 1 h at 0 °C with 25 µL of the labelled ligand as well as 25 µL of one competitor compound, of which eight different concentrations were tested per assay. “Total” and “Non-Specific” binding were determined in presence either of mQ water or unlabeled apamin (0.1 µM) respectively. At the end of the incubation period, the samples were filtered under reduced pressure. The incubation tubes were rinsed with 2.5 mL of ice-cold buffer. The filters were then rapidly washed with 2.5 mL of ice-cold buffer, twice, and placed in a vial containing 7.5 mL of scintillation cocktail (EcolitePlus, MPBio, Solon, OH, USA). Radioactivity was evaluated with a Packard Tri-Carb 2910TR Beta-counter (PerkinElmer, Shelton, CT, USA) after a minimum of 3 h of contact in absence of light. Curve fitting was carried out using the calculated specific binding values for each concentration of competitors using GraphPad Prism version 9.2 to obtain IC_50_ values. These data are then further transformed into inhibitory constant (K_i_) values using the Cheng-Prusoff equation with the radioligand’s K_D_ values corresponding to the target: 5.009 pM for SK2 and 6.576 pM for SK3 [1].

Data plotting and graphical curves were generated using GraphPad Prism (version 9.2; GraphPad Software, San Diego, CA, USA). Macromolecular structures and molecular graphics were visualized using PyMOL (version 3.1; Schrödinger, Inc., New York, NY, USA). 

### 3.3. In Silico Calculations

All calculations were performed using Maestro (Schrödinger Release 2024-3, Schrödinger, LLC, New York, NY, USA, 2024). Structures of the ligands have been generated using LigPrep taking care to assign the right protonation/ionisation state to the compound. Pharmacophore models were constructed using the Phase module in Maestro [2]. This procedure led to the development of a five-points pharmacophore. Compounds not used to generate the pharmacophore were fitted on the five-points pharmacophore using the “Ligand and database screening” tool.

## 4. Conclusions

A new pharmacophore model was generated using a series of bis-THIQ derivatives as a training set. The model successfully predicted the interaction of two recently identified alkaloids, **(1)** and **(2)**, which were not included in the pharmacophore generation process. The model was further used to predict the affinity of selected bis-THIQ alkaloids displaying diverse structural features, correctly identifying their binding potential with the unique exception of **(7)**.

This pharmacophore model therefore represents a valuable new tool for the rational design of small molecules targeting the apamin-sensitive site of SK channels. In particular, several bis-THIQ scaffolds identified in this study may contribute to a better understanding of the specific structural requirements of the THIQ scaffold for interacting with the apamin-binding site. However, like all computational tools, the model exhibits certain limitations; for instance, it failed to predict the behavior of **(7)**, which showed no experimental binding despite satisfying the model’s pharmacophoric criteria.

Five additional bisbenzylisoquinoline alkaloids, namely **(3)**, **(5)**, **(4)**, **(6)**, and **(7)**, were investigated in this study to further explore the interaction of this chemical family with SK channels. The evaluation of these structurally diverse compounds provided valuable insights into the structural determinants governing affinity for the apamin-sensitive site of SK channels. Notably, four of the five tested alkaloids displayed significant binding affinities, confirming the relevance of bis-THIQ scaffolds as promising ligands for these channels.

Moreover, the basic nature of these compounds represents an important advantage for future pharmacological applications, as basic small molecules generally exhibit favorable properties for crossing biological barriers. Consequently, these alkaloids serve as promising scaffolds for the development of orally bioavailable ligands.

## Figures and Tables

**Table 1:** Affinity of bis-THIQ alkaloids fitting the selected pharmacophore model for SK channel subtypes (Ki in µM; mean ± SD; number of independent experiments in parentheses with 8 concentrations evaluated in duplicate; * data from [3]).

Compounds		SK2 Affinity	SK3 Affinity	SK2 *Versus* SK3 Selectivity
Cyclic	Cepharanthine *	1.318 ± 0.281 (3)	1.091 ± 0.0919 (4)	0.83
	Oxyacanthine *	0.284 ± 0.066 (5)	0.679 ± 0.323 (4)	2.17
	Fangchinoline	0.576 ± 0.144 (4)	0.314 ± 0.084 (6)	0.55
	Cycleanine	>10	>10	-
Non-cyclic	Dauricine	4.8 ± 0.39 (6)	4.46 ± 0.33 (6)	0.93
	Isoliensinine	0.610 ± 0.203 (4)	0.462 ± 0.098 (4)	0.76
	Neferine	0.451 ± 0.122 (4)	0.754 ± 0.173 (6)	1.67

## Data Availability

The original contributions presented in this study are included in the article/Supplementary Materials. Further inquiries can be directed to the corresponding authors.

## Supplementary Materials

The following supporting information can be downloaded at: https://www.mdpi.com/article/10.3390/molecules31152723/s1, Table S1: List of pharmacophore models generates. Site types are defined as A = H-Bond acceptor, H = hydrophobic, R = aromatic, P = positive; Table S2: Affinity of bis-(1,2,3,4-tetrahydroisoquinolinium) stereoisomers and parent compounds for SK2 and SK3 channels used for the pharmacophore from [35]; Figure S1: Comparison of the three first hypothesis (AHHPR_1, AAHPR_2, and AHHPR_3) illustrating their similarity in terms of pharmacophore sites; Figure S2: Structural changes between the small molecule SK channel blocker AG525 E1 and the envisioned series of simplified analogues, designed to reduce the size and diastereomeric state of the dimeric AG525 E1 into a simpler enantiomeric series of lesser volume; Figure S3: Examples of configurations for three of the AG525-THIQ simplified-derivatives; Figure S4: Chemical structure of the molecules used for the construction of the pharmacophore; Refs. [4,35,71,72] are cited in the Supplementary Materials.
