# Supplementary material for: Further Investigations on Natural and Synthetic Bis-(1,2,3,4-tetrahydroisoquinoline) Derivatives Interacting with the Apamin-Sensitive Site of Small-Conductance Calcium-Activated Potassium Channels
Source: Molecules. 2026 Aug 5;31(15):2723. doi: 10.3390/molecules31152723 (PMC13468207; doi:10.3390/molecules31152723)
Supplement: Supplementary file 1 [file molecules-31-02723-s001.zip › molecules-4439425-supplementary.pdf]

Further investigations on natural and synthetic bis-(1,2,3,4-tetrahydroisoquinoline) derivatives interacting with the apamin-sensitive site of small-conductance calcium-activated potassium channels.

Hossein Taouba[1+], Romain Vitello[1+], Donna Pereira Barbon[1], Jean-Luc Hayen[1], Coralie Herbet[1], Szymon Faliński [1], Nikolay Tumanov[2], Johan Wouters[2], Jean-François Liégeois[1]*

Supplementary materials

Material and methods

Drugs

Compounds tested in this study were purchased from the following companies: fangchinoline (ABCR AB550543, 97 %), isoliensinine (ABCR AB493998, 95 %); dauricine BioConnect (TargetMol T6S0119, > 99 %); cycleanine (TargetMol T8206 > 99.5%), neferine (Sigma-Aldrich SML0349, ≥98%). They have been used without further purification. The simplified AG525 analogs purity range between 91% to 99%. The compounds were dissolved in DMSO at 40 x 10^{-4} M with sonication before the experiments.

Experimental procedures for cell culture and transfection, biological material preparation and affinity binding measurements

Cell culture

Media, complements and consumables were purchased from different companies (VWR, Fisher, Thermo Scientific, Sigma-Aldrich, Greiner, Biowest, Gibco, Falcon, Eppendorf).

HEK293 cells were used. This cell line was available in the laboratory and was maintained using Dubelco's Modified Eagle Medium (DMEM) containing high glucose, stable-glutamine and complemented with Foetal Bovine Serum (FBS) and a Penicillin-Streptomycin mix (100 U/L and 100 mg/L respectively) and was incubated at 37 °C and under 5 % CO_2 atmosphere. The cells were passaged twice a week with a 10 % dilution. To do so, cells were detached by 5 min of trypsinization at 37 °C and were then centrifuged at about 250 x g for 5 min at room temperature before resuspension in fresh medium. The HEK293 cells were kept for a maximum of 25 cycles.

Transient transfection

The cells were transiently transfected as needed using linear PolyEthyleneImine (M.W. 25000 Da) from Alfa Aesar (ref. 43896.01) with plasmids containing the KCNN2 and KCNN3 human genes bought from Genscript. The genes were inserted in a pcDNA3.1 plasmid in house. The complete plasmids were therefore available at the laboratory.

Biological material preparations

The cell line used in this study was generously provided by Professor Vincent Seutin from GIGA Neurosciences. The cells were originally obtained from LGC Standards Sarl (ATCC), and the specific reference for this cell line is **HEK 293 (CRL-1573)**.

HEK293 cells were plated on 100 mm Petri dishes for 24 to 48 h, until a confluence of about 90 % (75 % to 100 %) was reached. They were then transfected with plasmids containing either the hSK2 or the hSK3 gene, in a 0.2 µg/µL solution of PEI. Cells from 5 to 10 Petri dishes were harvested together after 48 h of contact by 5 min of trypsinization at 37 °C and concentrated by 5 min of centrifugation at about 250 x g at room temperature. The pellet was then resuspended in 3 ml per dish of cold PBS (4 °C) and concentrated by 10 min of centrifugation at 1000 x g at 4 °C. This PBS wash was repeated a second time. Afterwards, the pellet was resuspended in a lysis buffer (50 mM Tris, 1 % BSA, pH 7.4), mixed thoroughly using a 21G needle mounted on a syringe, and centrifuged at 200 x g for 10 min at 4 °C. The supernatant was gathered and centrifuged for 25 min at 20000 x g at 4 °C, the resulting pellet was then resuspended in a storage buffer (50 mM Tris, 5 mM KCl, pH 8.5) using 1 ml per dish. The resuspension in storage buffer and centrifugation was repeated a second time.

The protein concentration was determined using a colorimetric protein assay kit (Pierce BCA protein assay kit, Rockford, IL, USA at Thermo Scientific). The absorbance of the samples and of a reference scale was measured on transparent 96-well plates at 560 nm on a FlexStation3 apparatus. The concentration was calculated by linear regression using the scale. The preparation was then diluted with 10 % (v/v) glycerol and aliquots were stored at -80 °C before use.

Binding experiments

The incubation buffer and the wash buffer had the same composition in this procedure and consisted of 10 mM of Tris-HCl (pH 7.5), with 0.1 % (m/v) of BSA and 5 mM of KCl. The radioligand was ^{125}I-apamin, with a specific activity of 2000 to 2200 Ci/mmol, and was used at a concentration of about 5 pM in the assay. Unlabeled apamin was diluted in mQ water at a concentration of 400 µM as stock solution and aliquots were stored at -20 °C before use. Glass fiber filters (Whatman GF/C) were pre-soaked in a 0.5 % (m/v) PEI (Sigma-Aldrich P3143) solution and washed with 2.5 mL of ice-cold buffer right before filtration. A membrane preparation containing either cloned hSK2 or hSK3 channels at corresponding concentration of 10 µg of protein per mL was prepared in the buffer. Membrane

samples of 950 µL were then incubated for 1 h at 0 °C with 25 µL of the labelled ligand as well as 25 µL of one competitor compound, of which eight different concentrations were tested per assay. "Total" and "Non-Specific" binding were determined in presence either of mQ water or unlabeled apamin (0.1 µM) respectively. At the end of the incubation period, the samples were filtered under reduced pressure. The incubation tubes were rinsed with 2.5 ml of ice-cold buffer. The filters were then rapidly washed with 2.5 mL of ice-cold buffer, twice, and placed in a vial containing 7.5 mL of scintillation cocktail (EcolitePlus, MPBio). Radioactivity was evaluated with a Packard Tri-Carb 2910TR Beta-counter after a minimum of 3 hours of contact in absence of light. Curve fitting was carried out using the calculated specific binding values for each concentration of competitors using GraphPad Prism version 9.2 to obtain IC_{50} values. These data are then further transformed into inhibitory constant (K_i) values using the Cheng-Prusoff equation with the radioligand's K_D values corresponding to the target: 5.009 pM for SK2 and 6.576 pM for SK3 [1].

In silico calculations

All calculations were performed using Maestro (Schrödinger Release 2024-3, Schrödinger, LLC, New York, NY, 2024). Structures of the ligands have been generated using LigPrep taking care to assign the right protonation/ionisation state to the compound. Pharmacophore models were constructed using the Phase module in Maestro [2]. This procedure led to the development of a five-points pharmacophore. Compounds not used to generate the pharmacophore were fitted on the five-points pharmacophore using the "Ligand and database screening" tool.

Table S1. List of pharmacophore models generates. Site types are defined as A = H-Bond acceptor, H = hydrophobic, R = aromatic, P = positive.

Title	SiteTypes	Survival Score	Site Score	Vector Score	Volume Score	Selectivity Score	Adjusted Score
AHHPR_1	AHHPR	5.531	0.745	0.903	0.68	2.504	3.194
AAHPR_2	AAHPR	5.502	0.793	0.942	0.686	2.382	3.105
AHHPR_3	AHHPR	5.481	0.796	0.963	0.57	2.454	2.915
AAHPR_4	AAHPR	5.469	0.884	0.968	0.582	2.337	2.876
AHPRR_5	AHPRR	5.521	0.818	0.97	0.726	2.307	3.122
HHPRR_6	HHPRR	5.496	0.703	0.961	0.714	2.419	3.149
AAHPR_7	AAHPR	5.433	0.757	0.93	0.678	2.368	3.039
AHPRR_8	AHPRR	5.466	0.713	0.972	0.716	2.366	3.088
AAPRR_9	AAPRR	5.412	0.833	0.976	0.727	2.178	2.981
AHHPR_10	AHHPR	5.452	0.806	0.916	0.524	2.507	3.201
AHPR_11	AHPR	5.042	0.988	0.996	0.693	1.665	2.358
HPRR_12	HPRR	5.024	0.866	0.958	0.726	1.776	2.539
AHPR_13	AHPR	4.915	0.814	0.891	0.662	1.849	2.577
APRR_14	APRR	4.973	0.861	0.969	0.726	1.718	2.482
HHPR_15	HHPR	4.973	0.925	0.983	0.663	1.703	2.289
AHPR_16	AHPR	4.95	0.934	0.976	0.663	1.679	2.267
AAPR_17	AAPR	4.945	0.991	0.997	0.693	1.565	2.262
APRR_18	APRR	4.93	0.829	0.972	0.726	1.704	2.499
HPRR_19	HPRR	4.903	0.711	0.968	0.705	1.82	2.539
AHPR_20	AHPR	4.876	0.788	0.903	0.669	1.817	2.534

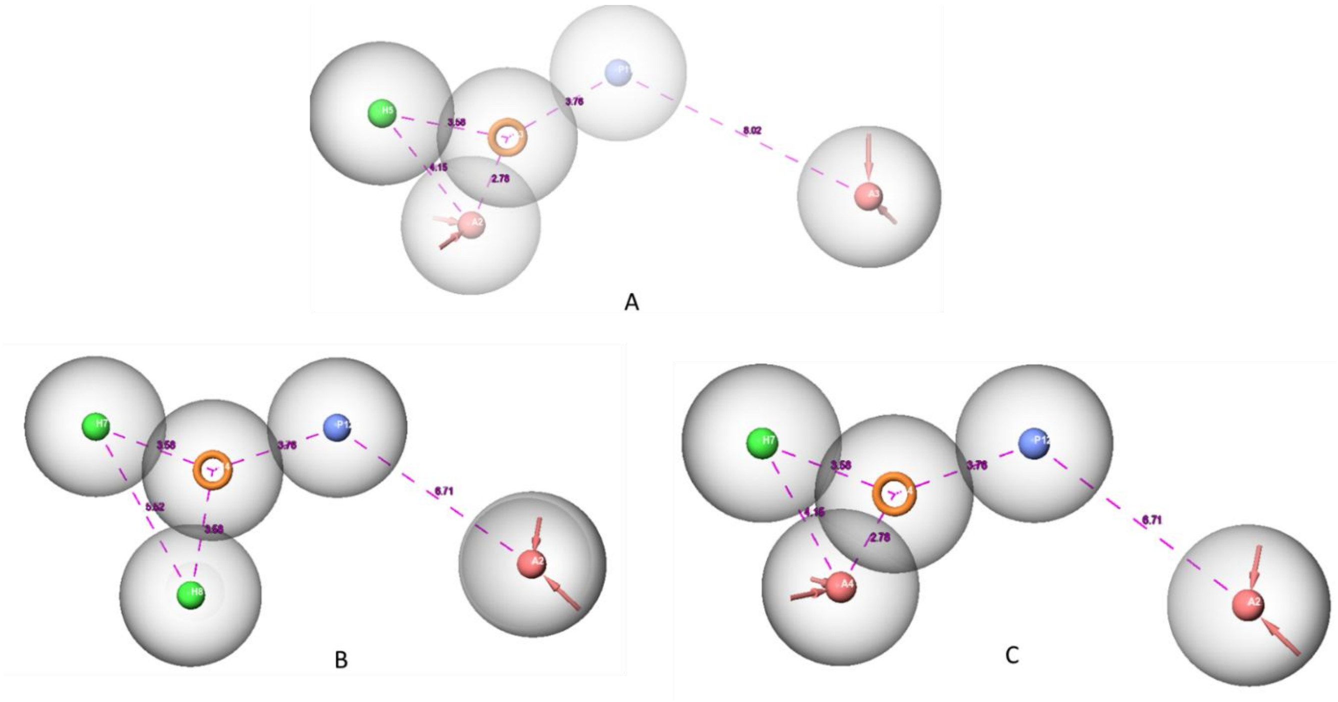

Figure S1. Comparison of the three first hypothesis (AHHPR_1, AAHPR_2, and AHHPR_3) illustrating their similarity in terms of pharmacophore sites. Site types are defined as A = H-Bond acceptor (red dot), H = hydrophobic (green dot), R = aromatic (orange cercle), P = positive (blue dot). Distances (Å) between the sites are added.

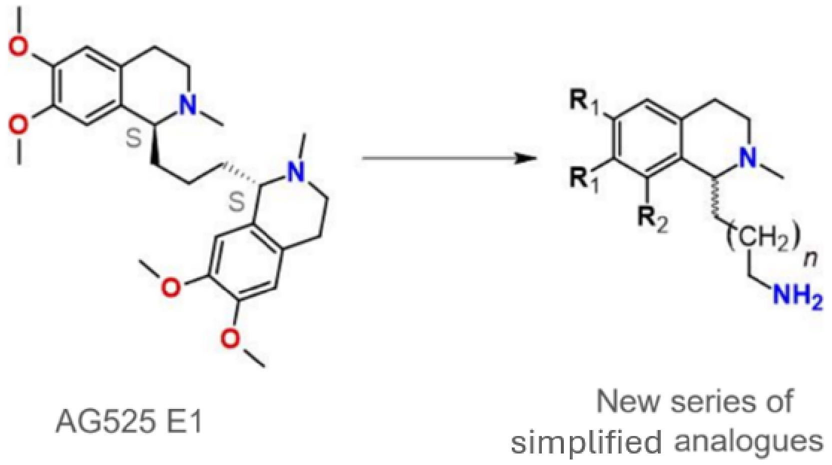

R_1	R_2	n
H	H	1
OMe	H	1
OMe	OMe	1
H	H	2
OMe	H	2
OMe	OMe	2
H	H	3
OMe	H	3
OMe	OMe	3

Figure S2: Structural changes between the small molecule SK channel blocker AG525 E1 and the envisioned series of simplified analogues, designed to reduce the size and diastereomeric state of the dimeric AG525 E1 into a simpler enantiomeric series of lesser volume [3].

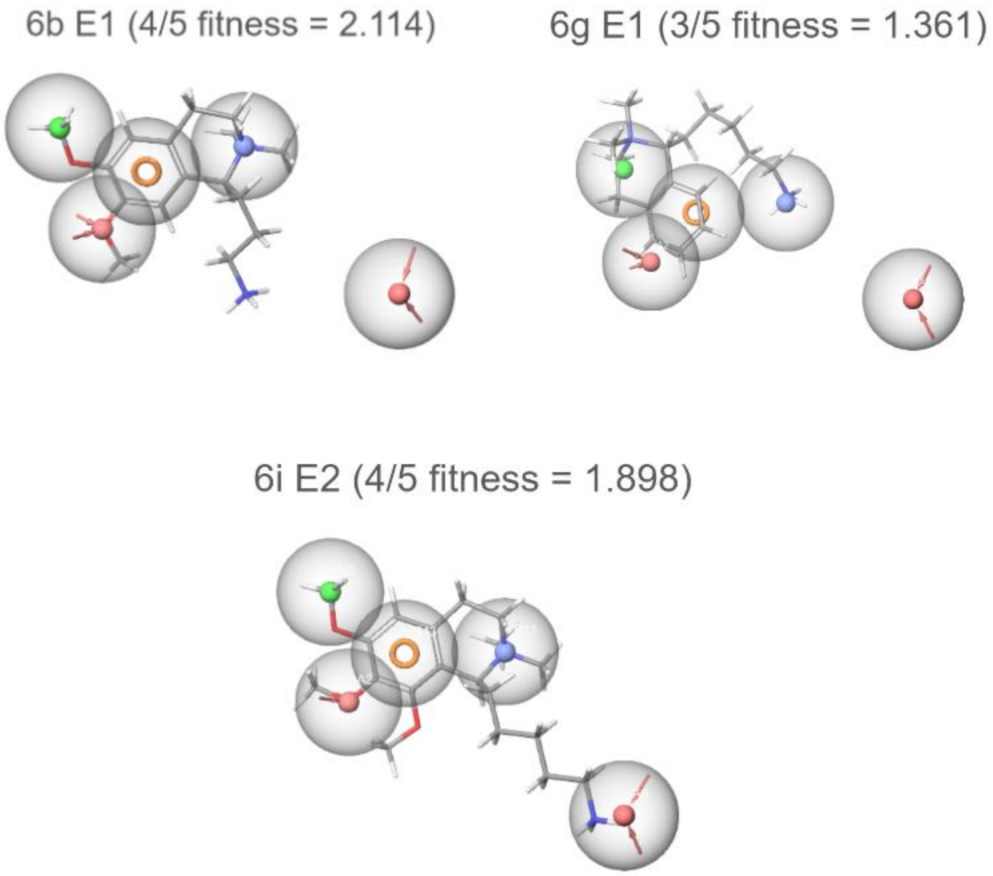

Figure S3: Examples of configurations for three of the AG525-THIQ simplified-derivatives tested with the proposed pharmacophore model [3].

The fitting of compound 6b (3-(6,7-dimethoxy-2-methyl-1,2,3,4-tetrahydroisoquinolin-1-yl)propan-1-amine) represents most of the series, missing one interaction point, the distal acceptor (A).

The fitting of compound 6i (5-(6,7,8-trimethoxy-2-methyl-1,2,3,4-tetrahydroisoquinolin-1-yl)pentan-1-amine) shows the side chain cannot fill the fifth interaction point.

The fitting of compound 6g (5-(2-methyl-1,2,3,4-tetrahydroisoquinolin-1-yl)pentan-1-amine) shows that non-methoxylated compounds can only fill three out of five interaction points.

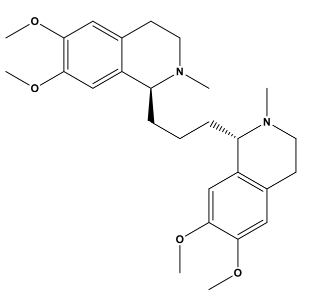

AG525E1

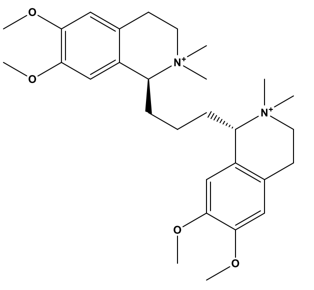

***N,N′* dimethyl-AG525E1**

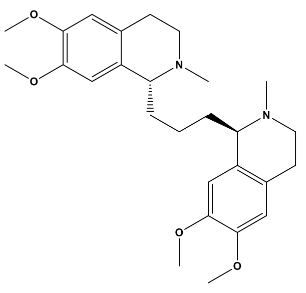

AG525E2

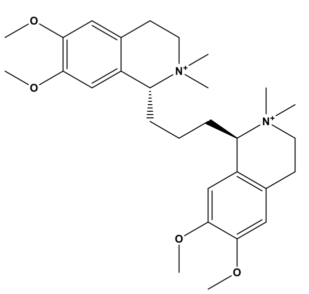

***N,N′* dimethyl-AG525E2**

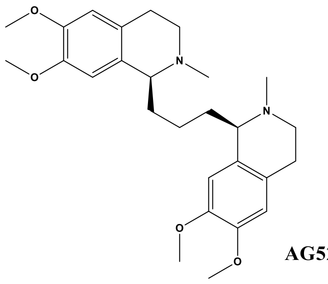

AG525Meso

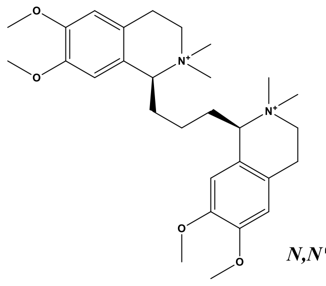

***N,N′* dimethyl-AG525Meso**

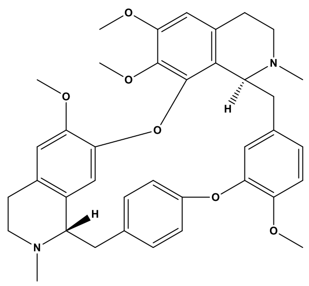

Tetrandrine

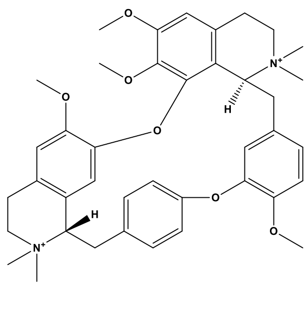

***N,N′* dimethyl-Tetrandrine**

Figure S4. Chemical structure of the molecules used for the construction of the pharmacophore.

Table S2. Affinity of bis-(1,2,3,4-tetrahydroisoquinolinium) stereoisomers and parent compounds for SK2 and SK3 channels used for the pharmacophore from [4].

	Compound	Scaffold	Absolute configuration	K_i [nM]	
				**SK2**	**SK3**
**1**	AG525E1	THIQ	*S,S*	276.5±40.6	370.1±93.5
**2**	AG525E2	THIQ	*R,R*	1255.8±170.3	1625±238
**3**	AG525 meso	THIQ	*S,R*/*R,S*	1685.3±149.4	1790.5±146.9
**4**	Tetrandrine	THIQ	*S,S*	305.5±49.8	461.3±42.3
**5**	*N,N'* dimethyl-AG525E1	NMTHIQ	*S,S*	51.6±7.9 (5.4)	77.2±1.2 (4.8)
**6**	*N,N'* dimethyl-AG525E2	NMTHIQ	*R,R*	65.7±5 (19.1)	162.7±21.8 (10)
**7**	*N,N'* dimethyl-AG525 meso	NMTHIQ	*S,R*/*R,S*	165.5±18.4 (10.2)	404.8±56.8 (4.4)
**8**	*N,N'* dimethyl-tetrandrine	NMTHIQ	*S,S*	40.5±1.5 (7.5)	90.3±20.4 (5.1)

References

1. Cheng Y-C, Prusoff WH Relationship between the inhibition constant (&) and the concentration of inhibitor which causes 50 percent inhibition (Iso) of an enzymatic reaction.

2. Dixon SL, Smondyrev AM, Rao SN (2006) PHASE: A Novel Approach to Pharmacophore Modeling and 3D Database Searching. Chem Biol Drug Des 67:370–372. https://doi.org/10.1111/j.1747-0285.2006.00384.x

3. Vitello R (2025) Towards better SK tools: improving the SK pharmacophore model. PhD. thesis ULiège

4. Liégeois J, Wouters J, Seutin V, Dilly S (2014) Bis-(1,2,3,4-tetrahydroisoquinolinium): A Chiral Scaffold for Developing High-Affinity Ligands for SK Channels. ChemMedChem 9:737–740. https://doi.org/10.1002/cmdc.201400028
